# Characterization of Oligometastatic Disease in a Real-World Nationwide Cohort of 3447 Patients With de Novo Metastatic Breast Cancer

**DOI:** 10.1093/jncics/pkab010

**Published:** 2021-02-04

**Authors:** Tessa G Steenbruggen, Michael Schaapveld, Hugo M Horlings, Joyce Sanders, Sander J Hogewoning, Esther H Lips, Marie-Jeanne T Vrancken Peeters, Niels F Kok, Terry Wiersma, Laura Esserman, Laura J van ‘t Veer, Sabine C Linn, Sabine Siesling, Gabe S Sonke

**Affiliations:** 1 Department of Medical Oncology, The Netherlands Cancer Institute, Amsterdam, the Netherlands; 2 Department of Epidemiology, The Netherlands Cancer Institute, Amsterdam, the Netherlands; 3 Department of Pathology, The Netherlands Cancer Institute, Amsterdam, the Netherlands; 4 Department of Research and Development, Netherlands Comprehensive Cancer Organisation, Utrecht, the Netherlands; 5 Department of Molecular Pathology, The Netherlands Cancer Institute, Amsterdam, the Netherlands; 6 Department of Surgical Oncology, The Netherlands Cancer Institute, Amsterdam, the Netherlands; 7 Department of Radiation Oncology, The Netherlands Cancer Institute, Amsterdam, the Netherlands; 8 Department of Surgical Oncology, University of California San Francisco, San Francisco, CA, USA; 9 Department of Laboratory Medicine, University of California San Francisco, San Francisco, CA, USA; 10 Department of Molecular Pathology, University Medical Center Utrecht, Utrecht, the Netherlands; 11 Department of Health Technology and Services Research, Technical Medical Centre, University of Twente, Enschede, the Netherlands; 12 Department of Clinical Oncology, University of Amsterdam, Amsterdam, the Netherlands

## Abstract

**Background:**

Observational studies in metastatic breast cancer (MBC) show that long-term overall survival (OS) is associated with limited tumor burden, or oligo-MBC (OMBC). However, a uniform definition of OMBC is lacking. In this real-world nationwide cohort, we aimed to define the optimal OMBC threshold and factors associated with survival in patients with OMBC.

**Methods:**

3535 patients aged younger than 80 years at diagnosis of de novo MBC in the Netherlands between January 2000 and December 2007 were included. Detailed clinical, therapy, and outcome data were collected from medical records of a sample of the patients. Using inverse-sampling-probability weighting, the analysis cohort (n = 3447) was constructed. We assessed OS according to number of metastases at diagnosis to determine the optimal OMBC threshold. Next, we applied Cox regression models with inverse-sampling-probability weighting to study associations with OS and progression-free survival in OMBC. All statistical tests were 2-sided.

**Results:**

Compared with more than 5 distant metastases, adjusted hazard ratios for OS (with 95% confidence interval [CI] based on robust standard errors) for 1, 2-3, and 4-5 metastases were 0.70 (95% CI = 0.52 to 0.96), 0.63 (95% CI = 0.45 to 0.89), and 0.91 (95% CI = 0.61 to 1.37), respectively. Ten-year OS estimates for patients with no more than 3 vs more than 3 metastases were 14.9% and 3.4% (*P *<* *.001). In multivariable analyses, premenopausal andperimenopausal status, absence of lung metastases, and local therapy of metastases (surgery and/or radiotherapy) added to systemic therapy were statistically significantly associated with better OS and progression-free survival in OMBC, independent of local therapy of the primary tumor.

**Conclusion:**

OMBC defined as MBC limited to 1-3 metastases was associated with favorable OS. In OMBC, local therapy of metastases was associated with better OS, particularly if patients were premenopausal or perimenopausal without lung metastases.

Observational studies in metastatic breast cancer (MBC) show that long-term survivors with MBC tend to present with a lower tumor burden at diagnosis, often referred to as oligometastases or oligometastatic breast cancer (OMBC) (1,2). Oligometastatic cancer is assumed to be a disease with limited widespread metastatic potential compared with widespread metastatic cancer and is therefore considered a favorable prognostic feature (3). A uniform definition of OMBC is lacking. Commonly, a maximum number of metastases, ranging from 1 to 5, is used as surrogate for potentially curable MBC (1,4‐6). However, it is hard to distinguish few metastases with limited metastatic capacity from few metastases that represent the tip of an iceberg of widespread, radiologically occult disease.

Based on the notion that patients with OMBC can achieve long-term remission, many such patients receive intensive therapy approaches including metastasectomies and stereotactic body radiotherapy (SBRT) (7‐13). Survival benefit, however, is derived from studies that are hampered by small numbers, single-institution data from secondary or tertiary referral centers, lack of adequate control groups, and limited follow-up. Because multimodality treatment can come with substantial toxicity, it is of utmost importance to determine which patients with limited MBC are likely to survive long-term and will derive benefit from such an approach. In the absence of randomized trial data in OMBC, we established a large, nationwide cohort of patients with de novo MBC (ie, patients who presented with distant metastases at first breast cancer diagnosis). We aimed to establish a definition for OMBC and study the impact of clinical factors and therapy on survival.

## Methods

### Patients

All patients aged younger than 80 years at diagnosis with de novo MBC between January 2000 and December 2007 were identified from the Netherlands Cancer Registry (NCR). The NCR is a nationwide cancer registry established in 1989 in the Netherlands and includes all breast cancer patients irrespective of stage at diagnosis or treatment (14). All basic clinical data in this study originated from the NCR, including age; menopausal status; tumor characteristics such as clinical primary tumor-regional lymph node-distant metastasis (TNM) and pathological TNM (pTNM) stage; estrogen receptor (ER) status, progesterone receptor (PR) status, HER2 status; and therapy details of the systemic and local therapy (surgery and/or radiation therapy) given for de novo MBC.

Trained registration clerks collected extensive additional clinical data from the medical records of all patients who survived more than 10 years after diagnosis and a matched sample of patients who did not (1 to approximately 3 frequency matched on ER status, age group, and year of diagnosis). Additional data included baseline performance status, comorbidities, number and detailed location of metastases, details of treatment, and first moment of progression since diagnosis of de novo MBC. For the number of metastases, the number of lesions was counted. Single-organ metastases was defined as metastases limited to 1 organ, regardless of number of lesions.

**Figure 1.**
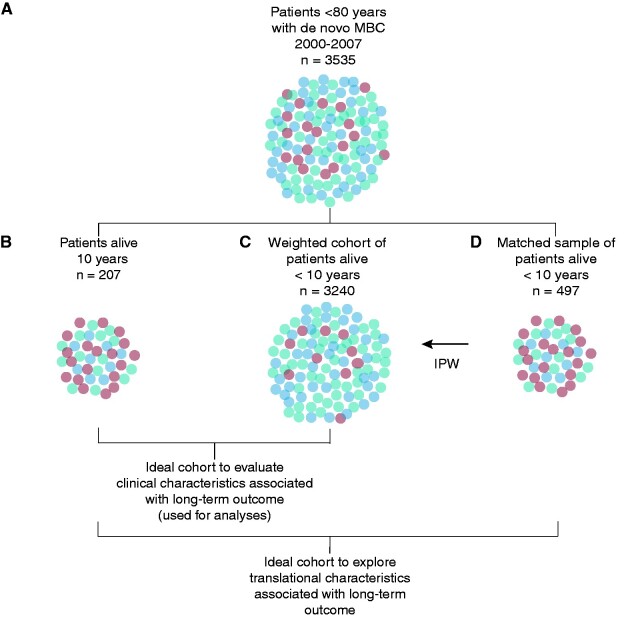
Schematic presentation of matched and weighted cohort. **A)** The population shown represents the complete population of patients diagnosed with metastatic breast cancer between 2000 and 2007 in the Netherlands. Dots are coded to illustrate the distribution of clinical characteristics, such as estrogen receptor status and age at diagnosis. **B)** Shows 5.9% of patients, who are alive more than 10 years since diagnosis. These patients have a different distribution of clinical characteristics. To allow exploration of biomarkers, we matched known clinical characteristics, which were ER status, age at diagnosis, and year of diagnosis, with a sample of patients not surviving 10 years **(D)**. To evaluate the effect of clinical characteristics and other known clinical characteristics, we calculated inverse-probability-weights based on the known distribution of ER, age groups at diagnosis, and year of diagnosis in the complete population **(A)** and the sample **(D)** to reconstruct the whole population **(C)**. Population **(B)** and **(C)** are used for the analyses. IPW = inverse probability weight; MBC = metastatic breast cancer.

Receptor status was complemented through linkage with the nationwide network and registry of histopathology and cytopathology in the Netherlands (PALGA) (15). ER and PR positivity were defined according to Dutch guidelines as more than 10% positive nuclear staining. HER2 positivity was defined as strong homogeneous membranous staining (3+ intensity) by immunohistochemistry or gene amplification by in situ hybridization in case of 2+ intensity by immunohistochemistry (16,17). Patients with missing treatment data were not excluded because this could be a result of short survival and therefore not “missing at random.” All treatments were based on physician’s choice and varied across patients, therefore we refrained from imputing treatment data.

This study was approved by the review board of the NCR and conducted in accordance with the Declaration of Helsinki. The review board of the NCR has declared that no informed consent was required for collection of the data.

### Statistical Analyses

To enable analysis of clinical characteristics in the complete cohort of patients with MBC with a representative distribution of matching factors, we calculated the inverse-probability sample weight (IPW) based on year of diagnosis, age categories, and ER status for all patients (see Figure 1) (18). IPWs were used to adjust the partial likelihood function for patients sampling and allow for a correct representation of the variables in the constructed complete cohort. No weight could be assigned for patients younger than 40 years of age diagnosed in 2000, and therefore, 36 patients were not represented in the analyses.

Our main endpoint was overall survival (OS). Vital status is annually updated via linkage with the Dutch Personal-Records Database. OS was calculated as time from MBC diagnosis until death (irrespective of cause) or censored at date of linkage, which was January 31, 2020 (19). Secondary endpoint was progression-free survival (PFS) and was defined according to Standardized Definitions for Efficacy End Points (STEEP) criteria (ie, time between MBC diagnosis till progression of disease, death due to any cause, or censored at last visit) (19).

To determine which of the commonly used thresholds for OMBC was associated with better OS (≤1, ≤3, or ≤5 metastases), we compared patients with 1, 2-3, and 4-5 metastases with patients with multiple (>5) metastases as a reference. Corresponding adjusted hazard ratios (HR) were based on multivariable Cox regression models, accounting for factors associated with OS in all patients at a *P *value less than* *.10 and factors differently expressed between groups. The OMBC threshold found by this method was used for further analyses.

Further analyses were focused on identifying prognostic factors in patients with OMBC. Factors associated with OS at a *P *value less than* *.10 in Cox regression models, adjusting for age and using IPW, were included in a multivariable Cox regression model. Adjusted hazard ratios and corresponding 95% confidence intervals (CIs) based on robust standard errors are reported. Ten-year OS estimates were calculated with the Kaplan-Meier method (20). All reported *P* values were 2-sided, and *P* values less than .05 were considered statistically significant. All statistical analyses were performed using Stata version 15.0.

### Sensitivity Analyses

We performed several sensitivity analyses. First, to evaluate the influence of the OMBC threshold on the association of local therapy of metastases with outcome, we performed a sensitivity analyses using the thresholds of no more than 1 metastasis and no more than 5 metastases to define OMBC. Second, a sensitivity analysis was performed by excluding patients aged 70 years or older at diagnosis to determine the influence of older age and factors associated with that which could have influenced care management and/or outcome.

Furthermore, we performed sensitivity analyses to evaluate the impact of immortal-time bias on the association between local therapy of the primary tumor, distant metastases, and outcome. In these sensitivity analyses, only local therapy of the primary tumor and local therapy of metastases performed within 200 days after diagnosis of OMBC were included in the model; 200 days was used to allow local therapy administered after upfront systemic therapy.

Because trastuzumab became widely available for patients with MBC after 2005 as first-line therapy, we performed another sensitivity analysis in patients diagnosed with MBC after 2005 to determine the effect of the availability of trastuzumab as first-line treatment.

Last, the proportional hazard assumption tested using Schoenfeld residuals and visual inspection (21) was violated for presence of bone metastases as survival curves crossed at approximately 5.5 years. We therefore performed a sensitivity analysis using an interaction with time at 5.5 years for bone metastases, estimating the hazard ratio separately for less than 5.5 years and 5.5 years or more of follow-up in the multivariable model (20). This does not affect the IPW used in the models (22).

## Results

### Clinical Characteristics of All Patients

**Figure 2.**
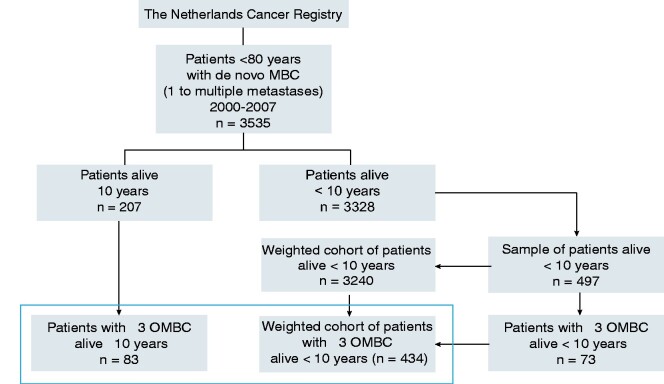
CONSORT diagram. Thebox indicates the analysis cohort of patients with OMBC (n=517). For all sampled patients surviving less than 10 years, the inverse probability sample weight was calculated based on year of diagnosis, age categories (20-39, 40-49, 50-59, 60-69, and 70-79 years), and ER status. Patients alive 10 years or more had a sample weight of 1. MBC = metastatic breast cancer.

**Figure 3.**
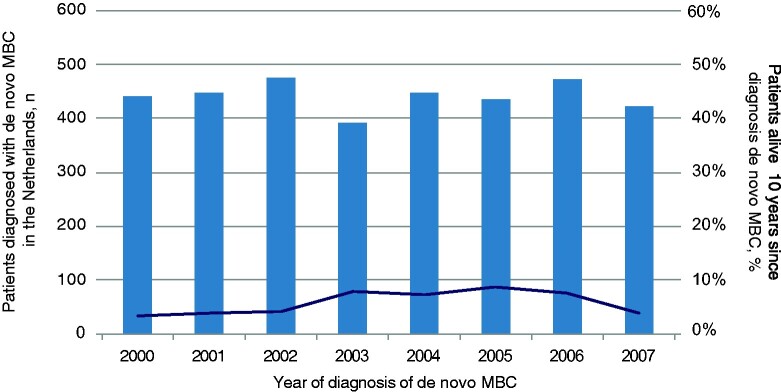
Between 2000 and 2007, on average, 5.9% of 3535 patients with de novo MBC survived 10 years or longer. The *x*-axis shows the year of diagnosis; the left *y*-axis represents number of patients diagnosed with de novo MBC in the Netherlands younger than 80 years (bars); the right *y*-axis represents the percentage of patients surviving at least 10 years (**darksolid line**). MBC = metastatic breast cancer.

Between 2000 and 2007, 3535 patients younger than 80 years of age developed de novo MBC (ie, patients who presented with distant metastases at first breast cancer diagnosis) in the Netherlands, of whom 207 (5.9%) were alive after 10 years (Figure 2). The incidence of de novo MBC remained stable over the inclusion period (Figure 3). The proportion of patients with MBC who were still alive 10 years later varied between 3.0% and 6.9%. Median follow-up was 15.2 years (interquartile range = 13.9-17.5); 96 patients died after 10 years. Pathological evidence of distant metastases was available in about one-third of patients, and two-thirds were based on imaging only.

The IPW cohort was based on 704 patients and represented 3447 patients (Figure 2). Baseline and treatment characteristics for patients grouped by number of metastases in the weighted cohort are shown in Supplementary Table 1 (available online). In patients with 1 metastasis, the lesion was less often located in the bones or lungs compared with patients with more metastases. Other baseline characteristics were comparable between the groups (Supplementary Table 1, available online). In a multivariable Cox regression model that included baseline characteristics, the number of metastases was statistically significantly associated with OS (Table 1). Compared with more than 5 metastases, the adjusted hazard ratio for OS in patients with 1, 2-3, and 4-5 metastases were 0.70 (95% CI = 0.52 to 0.96), 0.63 (95% CI = 0.45 to 0.89), and 0.91 (95% CI = 0.61 to 1.37), respectively. We therefore defined OMBC as no more than 3 distant metastases.

**Table 1.** Association of number of metastases with overall survival

Cutoff oligo	No. (%)^a^	10-y overall survival estimate^b^	Adjusted HR (95% CI)^c^	*P*
1 metastasis	269 (8.6)	17.1%	0.70 (0.52 to 0.96)	.03
2-3 metastases	248 (7.9)	12.5%	0.63 (0.45 to 0.89)	.009
4-5 metastases	95 (3.0)	7.4%	0.91 (0.60 to 1.37)	.65
>5 metastases	2528 (80.1)	3.2%	Referent	

a Numbers are based on the weighted cohort; the number of metastases was available for 3140 out of 3447 MBC patients. CI = confidence interval; HR = hazard ratio.

b Ten-year overall survival estimates are based on an univariable model.

c Hazard ratios are adjusted for age at diagnosis of MBC, breast cancer subtype, single-organ metastases, bone, liver, lung, and central nervous system metastases. The 95% confidence interval is based on robust standard errors.

### Clinical Characteristics of Patients With OMBC

Of the patients in the IPW cohort, 517 were diagnosed with 1-3 distant metastases (Figure 2). Baseline characteristics for these patients are shown in Table 2. Of these patients, 375 (72.5%) received endocrine therapy, and 269 (52.0%) received chemotherapy. Another 32 (6.2%) patients received unspecified systemic therapy. Of the patients, 215 (41.6%) received local therapy (surgery [n = 125], radiotherapy [n = 30], or a combination [n = 60]) of the primary tumor, and 124 (24.0%) patients received local therapy for metastases (either SBRT [n = 104], metastasectomy [n = 15], a combination of surgery and SBRT [n = 4], or thermal ablation [n = 1]). All but 1 patient who received local therapy for metastases also received systemic therapy. In 56 (44.4%) patients who received local therapy of metastases, this was combined with local therapy of the primary tumor.

**Table 2.** Baseline characteristics of the weighted cohort of patients with oligometastatic breast cancer (≤3 metastases) (n = 517)^a^

Characteristics	All patients with OMBC No. (%)
Year of diagnosis	
2000	43 (8.3)
2001	15 (2.9)
2002	68 (13.2)
2003	80 (15.5)
2004	70 (13.5)
2005	90 (17.4)
2006	60 (11.6)
2007	91 (17.6)
Age at diagnosis of MBC, y	
20-39	34 (6.6)
40-59	298 (57.6)
60-79	185 (35.8)
Menopausal status	
Premenopausal	121 (23.4)
Perimenopausal	81 (15.6)
Postmenopausal	315 (60.9)
Comorbidities	
No comorbidity	289 (55.9)
Single comorbidity	98 (19.0)
Multiple comorbidities	79 (15.3)
Unknown	51 (9.9)
Subtype	
ER+, HER2-/unknown	315 (60.9)
HER2+, ER+	63 (12.2)
HER2+, ER-	66 (12.7)
Triple-negative	36 (7.0)
Unknown	37 (7.2)
Single-organ metastases	
Yes	427 (82.6)
No	90 (17.4)
Location of metastases	
Lymph node metastases	39 (7.5)
Bone metastases	289 (55.9)
Liver metastases	176 (34.0)
Lung metastases	54 (10.4)
Skin metastases	14 (2.7)
CNS metastases	29 (5.6)
Diagnosis basis	
Radiological images	253 (48.9)
Radiological images and pathological evaluation	249 (48.2)
Unknown	15 (2.9)

a Percentages are based on known values unless unknown values are mentioned. ER = estrogen receptor; CNS = central nervous system; MBC = metastatic breast cancer; OMBC = oligometastatic breast cancer.

### Associations With Overall Survival and Progression-Free Survival in OMBC (≤3 Metastases)

**Figure 4.**
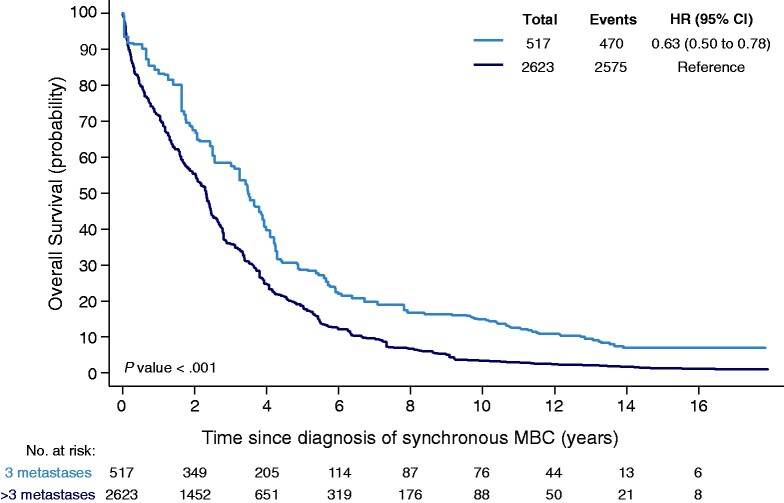
The 10-year OS estimate of patients with no more than 3 metastases is 14.9% vs 3.4% for patients with more than 3 metastases. Univariable hazard ratio is reported, and the 95% confidence interval is based on robust standard errors. Numbers of patients are based on the weighted cohort of patients. Number of metastases was known for 3140 patients. CI = confidence interval; HR = hazard ratio.

The 10-year OS estimate for patients with no more than 3 metastases was 14.9% vs 3.4% for patients with more than 3 metastases (*P *<* *.001; Figure 4), based on the weighted cohort. Factors independently associated with better OS in patients with OMBC included premenopausal and perimenopausal status, absence of lung metastases, and local therapy of metastases and the primary tumor (Table 3; Supplementary Figure 1, A-D, available online). Single-organ metastases was not independently associated with better OS. In comparison, local therapy of metastases was not associated with better OS in all patients with MBC (Supplementary Table 2, available online).

**Table 3.** Multivariable model of associations with overall survival in a weighed cohort of patients with oligometastatic breast cancer (≤3 metastases)

Characteristic	Adjusted hazard ratio (95% CI)^a^	*P*
Age (continuously)	1.00 (0.97 to 1.03)	.88
Menopausal status		
Premenopausal	0.37 (0.19 to 0.72)	.004
Perimenopausal	0.48 (0.25 to 0.91)	.03
Postmenopausal	Referent	
Breast cancer subtype		
ER+, HER2-/unknown	Referent	
HER2+, ER+	1.21 (0.62 to 2.36)	.57
HER2+, ER-	1.14 (0.53 to 2.44)	.74
Triple-negative	1.03 (0.47 to 2.27)	.94
Unknown	1.66 (0.64 to 4.31)	.30
Single-organ metastases		
Yes	1.23 (0.72 to 2.11)	.44
No	Referent	
Lung metastases		
Yes	4.83 (2.17 to 10.75)	<.001
No	Referent	
Bone metastases		
Yes	0.97 (0.56 to 1.67)	.91
No	Referent	
Skin metastases		
Yes	0.32 (0.04 to 2.64)	.29
No	Referent	
Systemic therapy^b^		
ET	Referent	
Taxane-based therapy +/- ET	1.17 (0.52 to 2.65)	.71
Taxane + anthracycline-based therapy +/- ET	0.99 (0.40 to 2.48)	.99
Anthracycline-based therapy +/- ET	1.44 (0.81 to 2.57)	.21
Other therapy +/- ET	1.12 (0.50 to 2.51)	.79
No systemic therapy	8.75 (2.11 to 28.54)	.002
Local therapy primary tumor^c^		
Yes	0.58 (0.37 to 0.89)	.01
No	Referent	
Local therapy metastases^d^		
Yes	0.57 (0.36 to 0.90)	.02
No	Referent	

a 95% confidence interval is based on robust standard errors. CI = confidence interval; CNS = central nervous system; ER = estrogen receptor; ET = endocrine therapy; MBC = metastatic breast cancer.

b A total of 484 patients received systemic therapy.

c Local therapy of the primary tumor was either (surgery n = 125, radiotherapy n = 30, or a combination n = 60). 90% of patients who received local therapy of the primary tumor also received systemic therapy.

d Local therapy of metastases was either stereotactic body radiotherapy (n = 104), metastasectomy (n = 15), a combination of surgery and stereotactic body radiotherapy (n = 4), or thermal ablation (n = 1). All but 1 patient who received local therapy of metastases also received systemic therapy. In 56 (44.4%) of the patients who received local therapy of metastases, the therapy was combined with local therapy of the primary tumor.

In patients with OMBC, the same factors were associated with better PFS as with OS: premenopausal and perimenopausal status, absence of lung metastases, local therapy of metastases, and local therapy of the primary tumor (Supplementary Table 3, available online).

### Sensitivity Analyses

Sensitivity analyses using no more than 1 and no more than 5 metastases as cutoff for OMBC were performed to evaluate the effect of local therapy of metastases. In patients with a solitary metastasis, the adjusted hazard ratio for OS for local therapy of metastases was similar but not statistically significantly associated with better OS (adjusted HR = 0.54, 95% CI = 0.29 to 1.04; *P *=* *.07), likely because of a limited number of events. In patients with no more than 5 metastases, the association of local therapy of metastases with OS was not statistically significant (adjusted HR = 0.67, 95% CI = 0.42 to 1.06; *P = *.09). The sensitivity analysis limited to patients aged younger than 70 years (n = 361) at diagnosis of OMBC was similar to the overall analysis (data not shown).

To reduce immortal-time bias, we performed a sensitivity analysis in which local therapy of metastases administered more than 200 days since diagnosis was not taken into account, which was the case in 34 of 124 patients. In this analysis, the association with better OS was less strong and no longer reached statistical significance (adjusted HR = 0.69, 95% CI = 0.41 to 1.16; *P = *.16). Using the same 200-day cutoff for local therapy of the primary tumor (22 of 215 patients), the association with better OS was also less strong and not statistically significant (adjusted HR = 0.71, 95% CI = 0.47 to 1.07; *P = *.10).

Limiting the analysis to patients diagnosed with OMBC between 2005 and 2007 (n = 240) when first-line trastuzumab became readily available in the Netherlands showed a favorable association with OS for trastuzumab treatment (adjusted HR = 8.23 x 10^-9^, 95% CI = 4.07 x 10^-9^ to 1.67 x 10^-8^; *P *<* *.001).

Because the proportional hazard assumption was violated for bone metastases, a last sensitivity analysis was performed incorporating an interaction with time for bone metastases. This showed a favorable association of bone metastases with OS in the first 5.5 years after diagnosis and an unfavorable association with OS after 5.5 years, both not statistically significant. The associations of other variables with OS were not affected.

The results of sensitivity analyses for PFS were similar to OS sensitivity analyses (data not shown).

## Discussion

The concept of oligometastatic cancer has received considerable attention in the oncologic literature. Two distinct scenarios are hypothesized to underlie the clinical phenomenon of oligometastatic cancer: a patient can either have widespread micrometastatic disease that goes largely undetected or a patient truly has 1 or only a few distinct distant metastases without further dissemination of cancer cells. The former resembles a patient with overt widespread disease in whom palliative systemic therapy may prolong survival and improve quality of life, but treatment is unlikely to offer cure, whereas the latter situation may call for a multimodality treatment approach including systemic therapy and radical treatment of distant metastases. The likelihood that systemic therapy will eradicate all different tumor clones present in a patient, similar as known in testicular cancer and Hodgkin lymphoma (23,24), is higher when the tumor burden is lower. In addition, the presence of only a limited number of metastases creates possibilities for local therapy. However, the current ability to distinguish among these various scenarios in individual patients is limited. We therefore aimed to define OMBC most likely to achieve long-term survival.

In this population-based study of patients with de novo MBC, patients with 1 or 2-3 metastases had better survival compared with patients with more than 5 metastases, whereas survival in patients with 4-5 metastases did not differ from those with more than 5 metastases. Characteristics that can help in selecting patients with OMBC most likely to achieve long-term survival include premenopausal and perimenopausal status and absence of lung metastases, because they were associated with better OS and PFS, after adjustment for age, breast cancer subtype, and therapy.

Some limitations should be acknowledged when interpreting the data of this study. First, patients in this cohort were diagnosed between 2000 and 2007, an era in which less advanced imaging techniques, local therapy techniques, and systemic treatment options were available. Less advanced imaging and therapy techniques will have resulted in a detection of less metastases than were actually present and less patients eligible for local therapy of metastases. Using no more than 3 metastases to define OMBC would not result in overtreatment of patients with limited MBC, because this comprises 16.5% of the metastatic population. We compared outcomes with patients with more than 5 metastases because radical treatment of all detected metastases if more than 5 would come along with increased risk of morbidity. Also, improved technical capability to treat more metastases locally does not necessarily translate into a survival benefit, and therefore, it is important to focus on patient selection. Future studies using advanced imaging and treatment will tell if extending the definition to 5 metastases similarly results in improved outcomes. Second, progress in systemic treatment options such as anti-HER2 therapies (25‐30), immune-checkpoint inhibitors (31‐33), and CDK4/6 inhibitors (34‐36) has improved outcome of patients with MBC. Most of these drugs were not available for patients in our cohort, and only 64.0% of patients with HER2-positive MBC received trastuzumab, of whom 54.4% as first-line therapy. A sensitivity analyses in patients who received trastuzumab as first-line treatment (those diagnosed since 2005) demonstrated a favorable association with outcome. The availability of other agents may change outcomes as well. However, given that there was a group that had statistically significantly better outcomes despite the lack of more targeted therapeutics does support the hypothesis that the biology of patients with 3 or less metastases is different, potentially amenable to cure. Third, using NCR registry data limited us to de novo MBC and availability of data in the registry and patient files. Data on response to systemic therapy was very limited. Furthermore, we do not know if the menopausal status is based on laboratory hormone levels or a physician’s note based on a rough estimation linked to age. Also, local and systemic therapies were not standardized but a reflection of physician’s choice based on patient and tumor characteristics and therefore subject to confounding by indication. We tried to reduce confounding by older age by excluding patients aged older than 80 years at diagnosis of MBC, because the treatment they received was not representative for all patients with MBC. Last, details on the exact dosages for SBRT and extent of surgery for metastases were incomplete. A potential pitfall of using IPW is unbalanced high weights for some patients with rare characteristics inducing less variety (37). However, this was not the case in our cohort and robust standard errors were used in all analyses. Last, in 48.2% of the patients with OMBC, 1 of the metastases was confirmed by pathologic evaluation. Considering the above, we were able to evaluate various thresholds used in clinical practice to define OMBC in a real-world cohort of patients with de novo MBC. We show that no more than 3 distant metastases is associated with improved OS, whereas patients with 4-5 distant metastases had similar OS to those with more than 5 distant metastases and may not benefit from local treatment of distant metastases.

We did not observe a favorable association between outcome and metastases limited to a single organ; however, we did see a favorable association with outcome for bone-only metastases (data not shown). Other studies have shown that single-organ metastases was favorably associated with survival in patients with OMBC (38) and MBC (29,39,40). These results might be influenced by a large number of patients having bone-only metastases. Single-organ involvement could also be another surrogate for less potential of metastatic spread and may therefore associate with better outcome, but our much larger study does not support using this characteristic.

In patients with OMBC, local therapy of metastases was associated with better outcomes. This is in line with observational and phase 2 studies on local therapy of OMBC (8‐12). Local therapy of metastases is thought to be beneficial because it eradicates a potential seeding source (41). It has the potential to cure OMBC if combined with systemic therapy, which is necessary to eradicate micrometastases, and local therapy of the primary tumor—if present. Almost all patients in our cohort who received local therapy of metastases also received systemic therapy. However, in 44.4% of patients who received local therapy of metastases, this was combined with local therapy of the primary tumor.

Local therapy of the primary tumor in patients with de novo MBC is subject of long, ongoing debate. Two meta-analyses showed an association with outcome and local therapy of the primary tumor (42,43). However, this finding has not been confirmed in randomized trials, including the recently presented ECOG-ACRIN-2108 (44‐46). Of note, these randomized trials evaluated local therapy of the primary tumor in the general MBC population; none of the trials focused on patients with OMBC and combined local therapy of the primary tumor with radical local therapy of all detected oligo-metastases, which could result in an OS benefit. However, the difference between observational and randomized studies might indicate that observational cohorts, including our study, demonstrate a benefit that is partly based on selection bias and immortal-time bias (5). When we excluded patients who received local therapy beyond 200 days (range = 204-491 days), the association between local therapy of the primary tumor and outcome was less strong and not statistically significant.

Besides using clinical characteristics to better define OMBC and select patients for a multimodality approach, we hypothesize that biomarkers such as circulating tumor cells (47), circulating tumor DNA (48), microRNAs (49‐51), and/or radiomics (52) have the potential to reveal more of the true biology underlying the few detected metastases. Four ongoing studies for patients with OMBC will evaluate the prognostic value of sequentially measured circulating tumor cells and/or circulating tumor DNA (NCT01706432, NCT02364557, NCT01646034, NCT03862911) (6).

In conclusion, in a real-world nationwide cohort of patients with de novo MBC, a maximum of 3 metastases appeared the optimal cutoff to define OMBC. The 10-year OS estimate of patients with OMBC is 14.9% compared with 3.4% in patients with more than 3 metastases. In patients with OMBC premenopausal and perimenopausal status, absence of lung metastases and local therapy of metastases were associated with better outcome.

## Funding

This work was supported by the Dutch Cancer Society/Pink Ribbon grant, grand ID: KWF 8216 Pink Ribbon 2016-212.

## Notes

**Role of the funder:** The sponsor had no role in in the design of the study; the collection, analysis, and interpretation of the data; nor in the writing of the manuscript or the decision to submit the manuscript for publication.

**Disclosures:** TGS has received funding from Memidis Pharma outside the current project. LE is an advisory board member for the Blue Cross Medical, an uncompensated board member of Quantum Leap Healthcare Collaborative, and received research support from Merck for an investigator-initiated trial for high-risk ductal carcinoma in situ (DCIS). LJV is a stock owner and employed (part-time) by Agendia NV, outside the scope of this article. SCL is an advisory board member for AstraZeneca, Cergentis, IBM, Pfizer, and Roche and received institutional research support from Agendia, AstraZeneca, Eurocept-pharmaceuticals, Genentech, Novartis, Pfizer, Roche, Tesaro, and Immunomedics. In addition, SCL received institutional nonfinancial support from Genentech, Novartis, Roche, Tesaro, and Immunomedics and other institutional support from AstraZeneca, Pfizer, Cergentis, IBM, and Bayer outside of this study. GSS has received institutional research funding from AstraZeneca, Merck, Novartis, and Roche, outside the current project. MS, HMH, JS, SJH, EHL, MJTVP, NFK, TW, and SS have no disclosures. GSS is principal investigator of the OLIGO-study (NCT01646034). TGS is the study coordinator of the OLIGO-study (NCT01646034). All other authors have declared no conflict of interest.

**Author contributions:** Study concepts and design: TGS, MS, SS, GSS, SCL. Financial support: TGS, EHL, SCL, SS, GSS. Administrative support: TGS, SH. Data gathering: SH. Quality control of data and algorithms: TGS, SH. Data analysis and interpretation: all authors. Statistical analyses: TGS, MS. Manuscript preparation: TGS. Manuscript editing: TGS, GSS. Manuscript review and approval: all authors.

**Acknowledgements:** The authors thank data managers of the Netherlands Comprehensive Cancer Organisation for the collection of data in the Netherlands Cancer Registry. We would also like to thank Rianne Hugen, Ingrid Prigge-Morsink, and Otto Visser for their assistance in gathering the additional clinical data. We thank PALGA for providing receptor status data.

## Data Availability

Interested investigators can request the data from the Netherlands Cancer Registry.

## Supplementary Material

pkab010_Supplementary_Data
