# Supplementary material for: Characterization of Oligometastatic Disease in a Real-World Nationwide Cohort of 3447 Patients With de Novo Metastatic Breast Cancer
Source: JNCI Cancer Spectr. 2021 Feb 4;5(3):pkab010. doi: 10.1093/jncics/pkab010 (PMC8099998; doi:10.1093/jncics/pkab010)
Supplement: pkab010_Supplementary_Data [file pkab010_supplementary_data.pdf]

SUPPLEMENTARY MATERIAL

Characterization of oligometastatic disease in a real-world nationwide cohort of 3,447 patients with de novo metastatic breast cancer

Tessa G Steenbruggen, Michael Schaapveld, Hugo M Horlings et al.

Supplementary Table 1. Comparison baseline and treatment characteristics between patients with 1 vs 2-3 vs 4-5 vs >5 metastases in the weighted cohort [a]

	patients with 1 metastasis (n = 269)		patients with 2-3 metastases (n = 248)		patients with 4-5 metastases (n = 95)		Patients with >5 metastases (n = 2,528)		*P* value [b]
	No.	(%)	No.	(%)	No.	(%)	No.	(%)	
Age at diagnosis of MBC									0.18
20-39	17	(6.3%)	18	(7.2%)	3	(3.2%)	148	(5.8%)	
40-59	129	(48.0%)	169	(68.1%)	35	(36.8%)	1,190	(47.1%)	
60-79	123	(45.7%)	61	(24.7%)	57	(60.0%)	1,190	(47.1%)	
Menopausal status									0.61
premenopausal	49	(18.2%)	72	(29.0%)	21	(22.1%)	461	(18.2%)	
perimenopausal	45	(16.7%)	37	(14.9%)	8	(8.4%)	498	(19.7%)	
postmenopausal	175	(65.1%)	139	(56.5%)	66	(69.5%)	1,569	(62.1%)	
Comorbidities									0.38
no comorbidity	135	(50.2%)	153	(61.7%)	38	(40.0%)	1,229	(48.6%)	
single	53	(19.7%)	45	(18.2%)	21	(22.1%)	632	(25.0%)	
multiple	65	(24.2%)	15	(6.0%)	11	(11.6%)	348	(13.8%)	
unknown	16	(5.9%)	35	(14.1%)	25	(26.3%)	319	(12.6%)	
Subtype									0.51 [c]
ER+, HER2-/unknown	176	(65.4%)	139	(56.0%)	54	(56.8%)	1,753	(69.3%)	
HER2+, ER+	28	(10.4%)	36	(14.5%)	16	(16.8%)	254	(10.1%)	
HER2+, ER-	37	(13.8%)	28	(11.3%)	25	(26.3%)	166	(6.6%)	
triple negative	18	(6.7%)	17	(6.9%)	0	(0%)	115	(4.5%)	
unknown	10	(3.7%)	28	(11.3%)	0	(0%)	240	(9.5%)	
Single-organ metastases									<0.001 [c]
yes	269	(100%)	158	(63.7%)	57	(60.0%)	1,149	(45.6%)	
no	0	(0%)	90	(36.3%)	38	(40.0%)	1,370	(54.4%)	
Location of metastases									
Lymph nodes	22	(8.2%)	17	(6.9%)	6	(6.3%)	370	(14.6%)	0.30
Bone	106	(39.4%)	183	(73.8%)	66	(69.5%)	2,008	(79.4%)	<0.001
Liver	92	(34.2%)	84	(33.9%)	38	(40.0%)	885	(35.0%)	0.98
Lung	4	(1.5%)	50	(20.2%)	20	(21.1%)	637	(25.2%)	0.006
Skin	14	(5.2%)	0	(0%)	0	(0%)	78	(3.1%)	0.52 [c]
CNS	13	(4.8%)	16	(6.5%)	0	(0%)	123	(4.9%)	0.84 [c]
Therapy									
First-line systemic therapy									0.08 [c]
endocrine therapy	111	(41.2%)	98	(39.5%)	44	(46.3%)	1,391	(55.0%)	
taxane-based therapy	30	(11.1%)	22	(8.9%)	0	(0%)	129	(5.1%)	
taxane+anthracycline based therapy	12	(4.5%)	13	(5.2%)	16	(16.8%)	64	(2.5%)	
anthracycline-based therapy	72	(26.8%)	94	(37.9%)	30	(31.6%)	624	(24.7%)	
other systemic therapy	11	(4.1%)	21	(8.5%)	5	(5.3%)	217	(8.6%)	
no systemic therapy	33	(12.3%)	0	(0%)	0	(0%)	103	(4.1%)	

Local therapy primary tumor [d]									0.10
local therapy	145	(54.0%)	70	(28.2%)	29	(30.5%)	863	(34.1%)	
no local therapy	124	(46.0%)	178	(71.8%)	66	(69.5%)	1,665	(65.9%)	
Local therapy metastases [e]									0.21
local therapy	70	(26.0%)	57	(23.0%)	10	(10.5%)	766	(30.3%)	
no local therapy	199	(74.0%)	191	(77.0%)	85	(89%.5)	1,762	(69.7%)	
Combination of treatments									<0.001
Local therapy all metastases and primary and systemic therapy	12	(4.4%)	1	(0.4%)	0	(0%)	0	(0%)	
Local therapy some metastases and primary and systemic therapy	27	(10.0%)	15	(6.0%)	0	(0%)	213	(8.5%)	
Local therapy all metastases and systemic therapy	1	(0.4%)	6	(2.4%)	0	(0%)	0	(0%)	
Local therapy some metastases and systemic therapy	28	(10.4%)	35	(14.1%)	9	(9.5%)	537	(21.5%)	
Local therapy primary and systemic therapy	75	(27.9%)	48	(19.4%)	28	(29.5%)	452	(18.1%)	
Systemic therapy only	93	(34.6%)	143	(57.7%)	58	(61.0%)	1,222	(48.9%)	
Local therapy primary	7	(2.6%)	0	(0%)	0	(0%)	7	(0.3%)	
Local therapy all metastases	1	(0.4%)	0	(0%)	0	(0%)	0	(0%)	
No therapy	25	(9.3%)	0	(0%)	0	(0%)	70	(2.8%)	

Abbreviations: ER, estrogen receptor; CNS, central nervous system; HER2, human epithelial growth factor receptor 2; MBC, metastatic breast cancer.

[a] Numbers and percentages are based on weighted cohort; the number of metastases was available for 3,140 out of 3,447 MBC patients. Percentages are based on known values unless unknown values are mentioned.

[b] P values are based on likelihood ratio test, unless stated otherwise.

[c] P value based on Pearson's Chi-squared test.

[d] Local therapy of metastases was either stereotactic body radiotherapy (26%), thermal ablation (<1%), surgery (1%) or both (1%).

[e] Local therapy of the primary tumor was either surgery (23%), radiotherapy (7%) or a combination (8%).

Supplementary Table 2. Multivariable model associations with OS in the weighted cohort consisting of all patients with de novo MBC

Characteristic	Hazard ratio (95% CI)	*P* value
Age (cont.)	1.01 (0.99 to 1.02)	0.35
Menopausal status		
premenopausal	0.70 (0.51 to 0.99)	0.04
perimenopausal	0.95 (0.70 to 1.28)	0.69
postmenopausal	reference	
Breast cancer subtype		
ER+, HER2-/unknown	reference	
HER2+, ER+	1.07 (0.83 to 1.37)	0.61
HER2+, ER-	1.23 (0.87 to 1.70)	0.22
triple-negative	2.01 (1.23 to 3.03)	0.002
unknown	1.92 (1.28 to 3.02)	0.004
Oligometastases (≤3 metastases)		
yes	0.67 (0.52 to 0.87)	0.002
no	reference	
Single-organ metastases		
yes	0.85 (0.66 to 1.05)	0.23
no	reference	
Bone metastases		
yes	0.97 (0.73 to 1.29)	0.85
no	reference	
Lung metastases		
yes	1.26 (0.96 to 1.65)	0.10
no	reference	
Liver metastases		
yes	1.44 (1.11 to 1.88)	0.006
no	reference	
CNS metastases		
yes	1.57 (1.01 to 2.44)	0.045
no	reference	
First-line systemic therapy		
ET	reference	
taxane-based therapy +/- ET	0.86 (0.61 to 1.22)	0.39
taxane+anthracycline-based therapy +/- ET	0.96 (0.56 to 1.66)	0.89
anthracycline-based therapy +/- ET	1.12 (0.87 to 1.44)	0.38
other therapy +/- ET	1.34 (0.96 to 1.89)	0.089
no systemic therapy	5.34 (2.86 to 9.95)	<0.001
Local therapy primary tumor [b]		
yes	0.82 (0.68 to 0.98)	0.03
no	reference	
Local therapy metastases [c]		
yes	1.03 (0.85 to 1.25)	0.78
no	reference	

Abbreviations: CI, confidence interval; CNS, central nervous system; ER, estrogen receptor; ET, endocrine therapy; HER2, human epithelial growth factor receptor 2, MBC; metastatic breast cancer.

[a] 95% confidence interval is based on robust standard errors.

[b] Local therapy of the primary tumor was either surgery, radiotherapy or a combination. See Table S1 for details.

[c] Local therapy of metastases was either stereotactic body radiotherapy, radiofrequency ablation or surgery.

Supplementary Table 3. Multivariable model of associations with PFS in weighted cohort of patients with de novo OMBC (≤3 metastases)

Characteristic	Adjusted hazard ratio (95% CI) [a]	*P* value
Age (cont.)	0.98 (0.95 to 1.02)	0.30
Menopausal status		
premenopausal	0.28 (0.14 to 0.57)	<0.001
perimenopausal	0.45 (0.24 to 0.83)	0.01
postmenopausal	reference	
Breast cancer subtype		
ER+, HER2-/unknown	reference	
HER2+, ER+	1.97 (0.99 to 3.92)	0.05
HER2+, ER-	1.89 (1.04 to 3.43)	0.04
triple-negative	0.57 (0.27 to 1.22)	0.15
unknown	1.71 (0.51 to 5.76)	0.39
Single-organ metastases		
yes	1.24 (0.51 to 2.20)	0.47
no	reference	
Lung metastases		
yes	3.82 (1.79 to 8.14)	0.001
no	reference	
Bone metastases		
yes	0.88 (0.52 to 1.51)	0.64
no	reference	
Skin metastases		
yes	0.14 (0.01 to 1.84)	0.14
no	reference	
First-line systemic therapy		
ET	reference	
taxane-based therapy +/- ET	0.57 (0.29 to 1.12)	0.10
taxane + anthracycline-based therapy +/- ET	0.48 (0.23 to 1.02)	0.06
anthracycline-based therapy +/- ET	1.16 (0.64 to 2.12)	0.62
other therapy +/- ET	0.79 (0.29 to 2.09)	0.63
no systemic therapy	14.28 (3.47 to 58.82)	<0.001
Local therapy primary tumor [b]		
yes	0.57 (0.37 to 0.88)	0.01
no	reference	
Local therapy metastases [c]		
yes	0.46 (0.29 to 0.73)	0.001
no	reference	

Abbreviations: CI, confidence interval; CNS, central nervous system; ER, estrogen receptor; ET, endocrine therapy; HER2, human epithelial growth factor receptor 2.

[a] 95% confidence interval is based on robust standard errors.

[b] Local therapy of the primary tumor was either (surgery n=125, radiotherapy n=30, or a combination n=60). 90% of patients who received local therapy of the primary tumor also received systemic therapy.

[c] Local therapy of metastases was either stereotactic body radiotherapy n=104, metastasectomy n=15, a combination of surgery and stereotactic body radiotherapy n=4 or thermal ablation n=1). All but one patient who received local therapy of metastases also received systemic therapy. In 56 of 126 (44.4%) patients who received local therapy of metastases, the therapy was combined with local therapy of the primary tumor. See Table S1 column 1 and 2 for details.

Figure S1

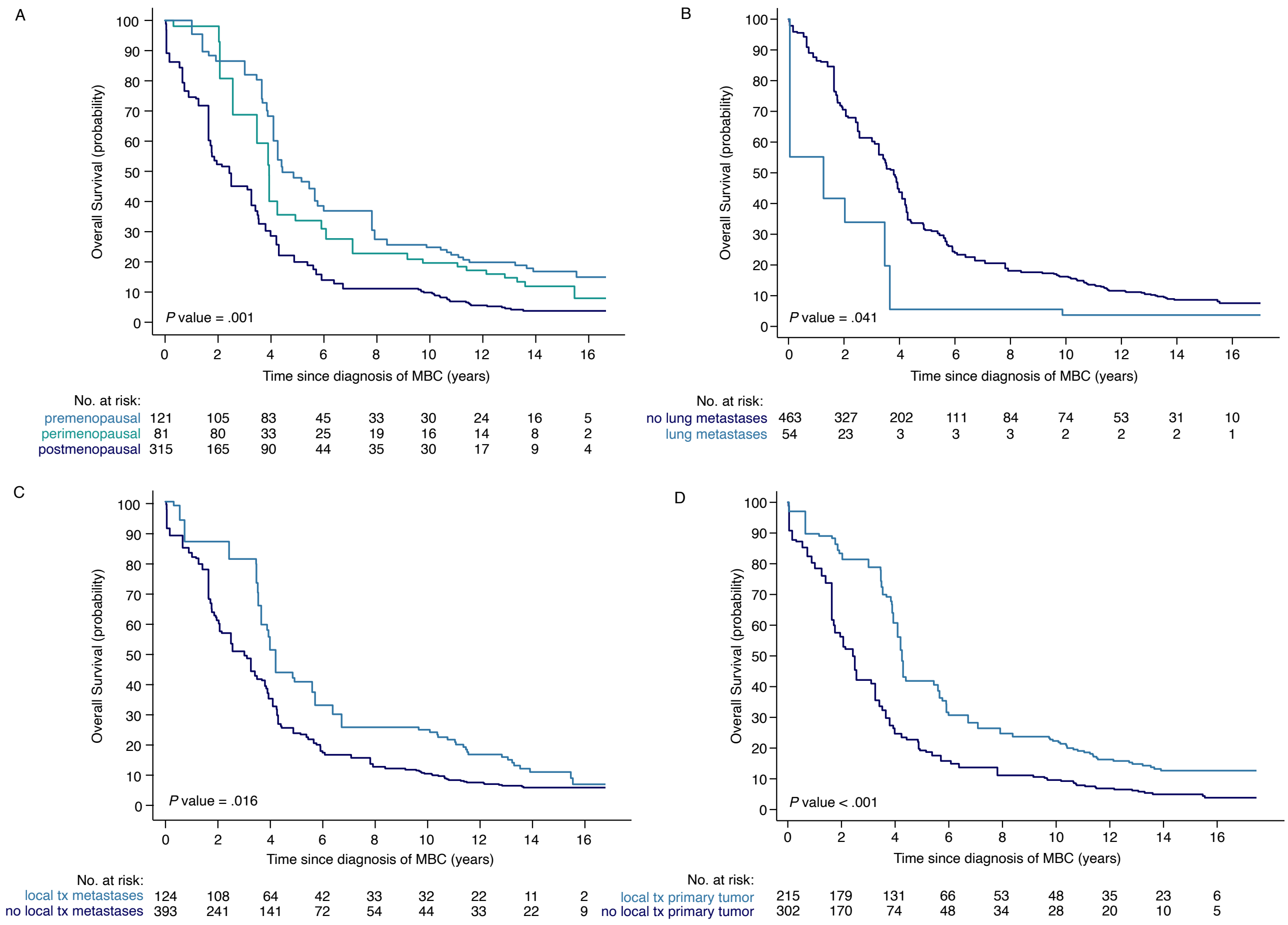

Supplementary Figure 1. Overall survival in patients with de novo oligometastatic breast cancer (≤3 metastases) per subgroup of patients (menopausal status, lung metastases, local therapy of metastases and local therapy of the primary tumor)

Local therapy of metastases was either stereotactic body radiotherapy, radiofrequency ablation or surgery. Local therapy of the primary tumor was either surgery, radiotherapy or a combination, see Table S1 column 1 and 2 for details. All but one patient who received local therapy of metastases also received systemic therapy. In 56 of 126 (44.4%) patients who received local therapy of metastases, the therapy was combined with local therapy of the primary tumor.
